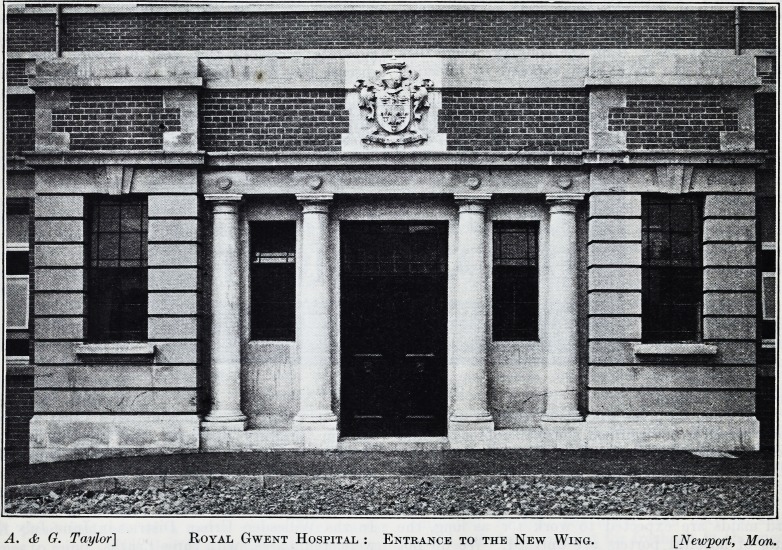# An Ambitious Hospital Scheme

**Published:** 1924-10

**Authors:** 


### AN AMBITIOUS HOSPITAL SCHEME.

### ENLARGING THE ROYAL GWENT.

The Royal Gwent Hospital, a striking E-shaped building, standing on the outskirts of Newport (Mon.), and providing treatment for the accident cases from the neighbouring docks and coal mines, as well as for the sick of a large town, has recently added to its usefulness by the opening of a new out-patients' department. Beginning eighty-five years ago as a small dispensary, the hospital steadily increased in size and efficiency. The dispensary became the Newport and County Infirmary, the Infirmary became the Newport and Monmouthshire Hospital, and that eventually developed in 1913 into the Royal Gwent Hospital, built on a site given by the late Viscount Tredegar.

*A. & G. Taylor*] Royal Gwent Hospital : Entrance to the New Wing. [*Newport, Mon.*

### New Out-Patients' Department.

It will be seen from the rapid development of the hospital that ample provision must be available for a large number of patients. For this reason a scheme has been projected for extensions to the hospital—at an estimated cost of £240,000—to provide wards, rooms for the staff, new kitchens, and an isolation block. The new out-patients' department is the first step towards the carrying out of this scheme. It is contained in a block which, like the rest of the hospital, is built of brick and Bath stone. This block has a large waiting hall on the ground floor and the out-patients' department and a ward on the first floor. This arrangement, which sounds odd, is a consequence of the peculiarities of the site owing to the slope of the ground. On passing through the entrance to the out-patients' department—an illustration of which we give—the patient comes first to the registrar's office in the vestibule, going thence to the large waiting hall, which gives access to the medical, surgical, ear, nose and throat, ophthalmic, and dental departments through small ante-rooms. The walls are lined with bands of marble of soft shades of green and grey.

### Space and Convenience.

For the convenience of the out-patients a buffet has been provided at the end of the hall. After treatment the patient goes along the exit corridor to the dispensary waiting-room, whence he leaves the building. The dispensary is adjoined by the dispenser's office and the out-patients' dispensary waiting-room, and is connected with the main corridor; grouped with it are a dispensary store and a class room for instructional purposes, the whole being arranged to give ample space and every convenience to patients and staff, besides being of a pleasing design. The architects are Messrs. Griggs & Vaughan, A.A.R.I.B.A., of Newport. The Royal Gwent Hospital's President is Lord Tredegar, and its Secretary Mr. J. K. Millward.

### Vale of Leven Cottage Hospital.

The Henry Brock Vale of Leven Cottage Hospital, founded through a bequest of £15,000 from the late Mr. Brock of Darleith, has been formally opened. The war delayed building, but, with interest, the trust fund grew to £20,546. On the advice of Col. Mackintosh, medical superintendent of the Western Infirmary, Glasgow, Broomley House was purchased and remodelled. Mr. Weddell was the architect. A balance of £10,000 is expected to remain as a nucleus of an endowment fund.

## Figures and Tables

**Figure f1:**